# Differential expression and localization of ADAM10 and ADAM17 during rat spermatogenesis suggest a role in germ cell differentiation

**DOI:** 10.1186/0717-6287-47-31

**Published:** 2014-07-04

**Authors:** Paulina Urriola-Muñoz, Carlos Lizama, Raúl Lagos-Cabré, Juan G Reyes, Ricardo D Moreno

**Affiliations:** Departamento de Fisiología, Pontificia Universidad Católica de Chile, Santiago, Chile; Cardiovascular Research Institute, University of California San Francisco, San Francisco, CA 94143 USA; Instituto de Química, Pontificia Universidad Católica de Valparaíso, Valparaíso, Chile; Departamento de Ciencias Fisiológicas, Facultad de Ciencias Biológicas, Pontificia Universidad Católica de Chile, Alameda 340, Santiago, Chile

**Keywords:** Testis, Spermatogenesis, Apoptosis, Paracrine, Metalloproteinase

## Abstract

**Background:**

Extracellular metolloproteases have been implied in different process such as cell death, differentiation and migration. Membrane-bound metalloproteases of the ADAM family shed the extracellular domain of many cytokines and receptor controlling auto and para/juxtacrine cell signaling in different tissues. ADAM17 and ADAM10 are two members of this family surface metalloproteases involved in germ cell apoptosis during the first wave of spermatogenesis in the rat, but they have other signaling functions in somatic tissues.

**Results:**

In an attempt to further study these two enzymes, we describe the presence and localization in adult male rats. Results showed that both enzymes are detected in germ and Sertoli cells during all the stages of spermatogenesis. Interestingly their protein levels and cell surface localization in adult rats were stage-specific, suggesting activation of these enzymes at particular events of rat spermatogenesis.

**Conclusions:**

Therefore, these results show that ADAM10 and ADAM17 protein levels and subcellular (cell surface) localization are regulated during rat spermatogenesis.

## Background

Mammalian testes have an intricate histology reflecting the complex interaction among different cell types, where para/juxtacrine cell signaling plays a fundamental role in order to produce a differentiate spermatozoa. Germ and Sertoli cells constitute the seminiferous epithelium, which is surrounded by a basal lamina and a layer of myoid cells [1]. Diploid germ cells (spermatogonia) are attached to the basal lamina, and they are a constantly dividing population that provides new cells that will eventually initiate meiosis [2]. Once germ cells undergoing meiosis (now named spermatocytes) reach the leptone stage they detach from the basal lamina, and from now on their survival and differentiation relies upon specific interaction with the Sertoli cell. Finally, as a product of meiosis, haploid cells (spermatids), will differentiate into a spermatozoon, which will detach from the seminiferous epithelium to the lumen of the seminiferous tubules and it will be store and become a fully mature cell in the epididymus. Within seminiferous tubules germ cells form concentric layers, where the differentiation wave goes from the basal lamina to the lumen of the seminiferous tubule [1]. Germ cells in adult rat testes can be found in 14 different cell associations or stages (numbered I-XIV), which can be distinguished according to the acrosome development, localization of elongated spermatids and meiosis steps [1, 3]. Several studies have shown that Sertoli cell morphology, seminiferous epithelium height, gene expression and protein localization are stage-specific suggesting that these particular associations reflect particular steps in germ cell differentiation [4–6].

Central in the juxta/paracrine communication is the shedding of growth factors or cytokines, which will initiate or end cell survival, differentiation or apoptosis. Transmembrane metalloproteinases of the ADAM (A Desintegrin And Metalloproteinase) family modulate the cell-cell and cell-matrix interaction, through the shedding of membrane-bound protein like grown factors, citokines, cell adhesion molecular and receptors [7, 8]. ADAM proteases are type 1 transmembrane proteins of approximately 70 to 120 kDa (mature proteins; the unprocessed precursors are about 20 kDa heavier due to their prodomain). They consist of a common modular ectodomain structure encompassing a variable transmembrane region; a cysteine-rich domain that can interact with cell surface proteoglycans and in some cases also contains a fusion peptide sequence; a disintegrin domain; a zinc-binding metalloprotease domain; and a prodomain that is cleaved off in the trans-Golgi network by protein convertases [9]. ADAMs metalloproteases are widely distributed throughout different tissues and development, and expression pattern and function of specific isoforms are tightly regulated during development, cell differentiation, insults and pathological conditions. In the male reproductive tract, ADAMs are differentially expressed in testes, epidydymis, efferent duct and prostate suggesting their function is regulated according to requirements of each organ [10].

We have previously shown that ADAM17 localize in germ cells undergoing apoptosis, and two different ADAM17 pharmacological inhibitors are able to prevent germ cell apoptosis, suggesting its pivoting role in this process [11]. In the same token, ADAM17 and ADAM10 are upregulated in pubertal rat testes after etoposide treatment, which induces apoptosis in germ cells [12, 13]. Pharmacological inhibition, *in vitro* and *in vivo* of both metalloproteases equally well prevent apoptosis, suggesting that activation of ADAM17 and ADAM10 is important during etoposide induced apoptosis in germ cells.

In order to better understand the physiological regulation of ADAM17 and ADAM10 during the differentiation process of germ cells, here we describe the localization and distribution of these metalloproteases during spermatogenesis in adult rats.

## Results

### Expression and localization of ADAM10 and ADAM17 in adult rat testes

**Figure 1:**
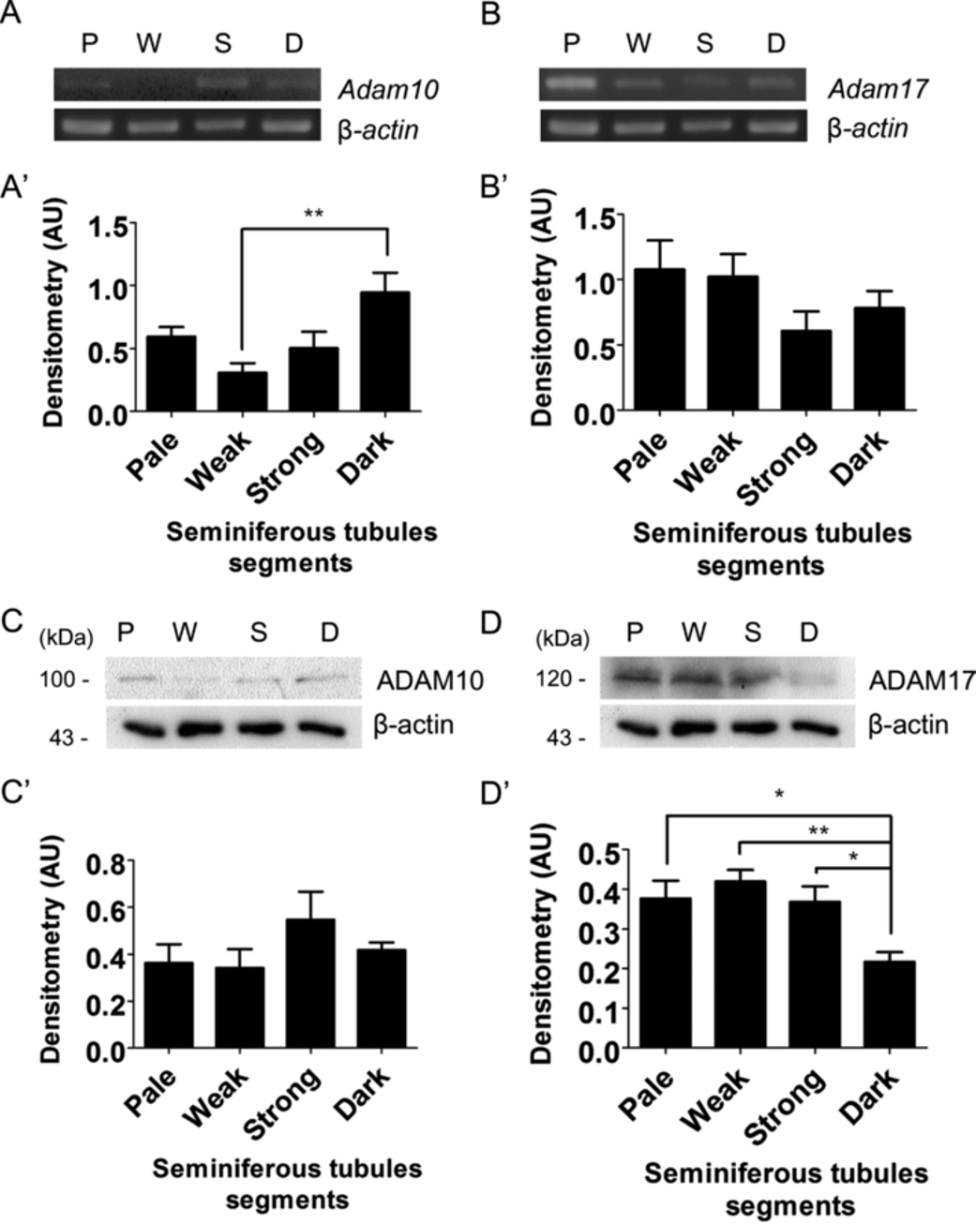
**mRNA and protein levels of ADAM10 and ADAM17 in different segments of rat seminiferous tubules.** *Adam10* **(A, A’)** and *Adam17* **(B, B’)** mRNA determined by semi-quantitative RT-PCR in Pale (P, stage IX-XII), Weak (W, stage XIII-I), Strong (S, stage II-V) and Dark (D, VI-VIII) segments using *B-actin* as a loading control. mRNA levels of *Adam10* were significantly higher in Dark than Weak segments, while *Adam17* levels did not change. ADAM10 **(C, C’)** and ADAM17 **(D, D’)** protein levels were determined by Western blotting **(C, D)** and then by densitometry using β-actin as a loading control. Protein levels of ADAM10 were similar in Pale (P, stage IX-XII), Weak (W, stage XIII-I), Strong (S, stage II-V) and Dark (D, VI-VIII) segments. On the other hand, ADAM17 protein levels significantly decreased in Dark as compared with the other segments. Bars represent the mean ± S.E.M. of three different experiments (*p < 0.001, **p < 0.01).

**Figure 2:**
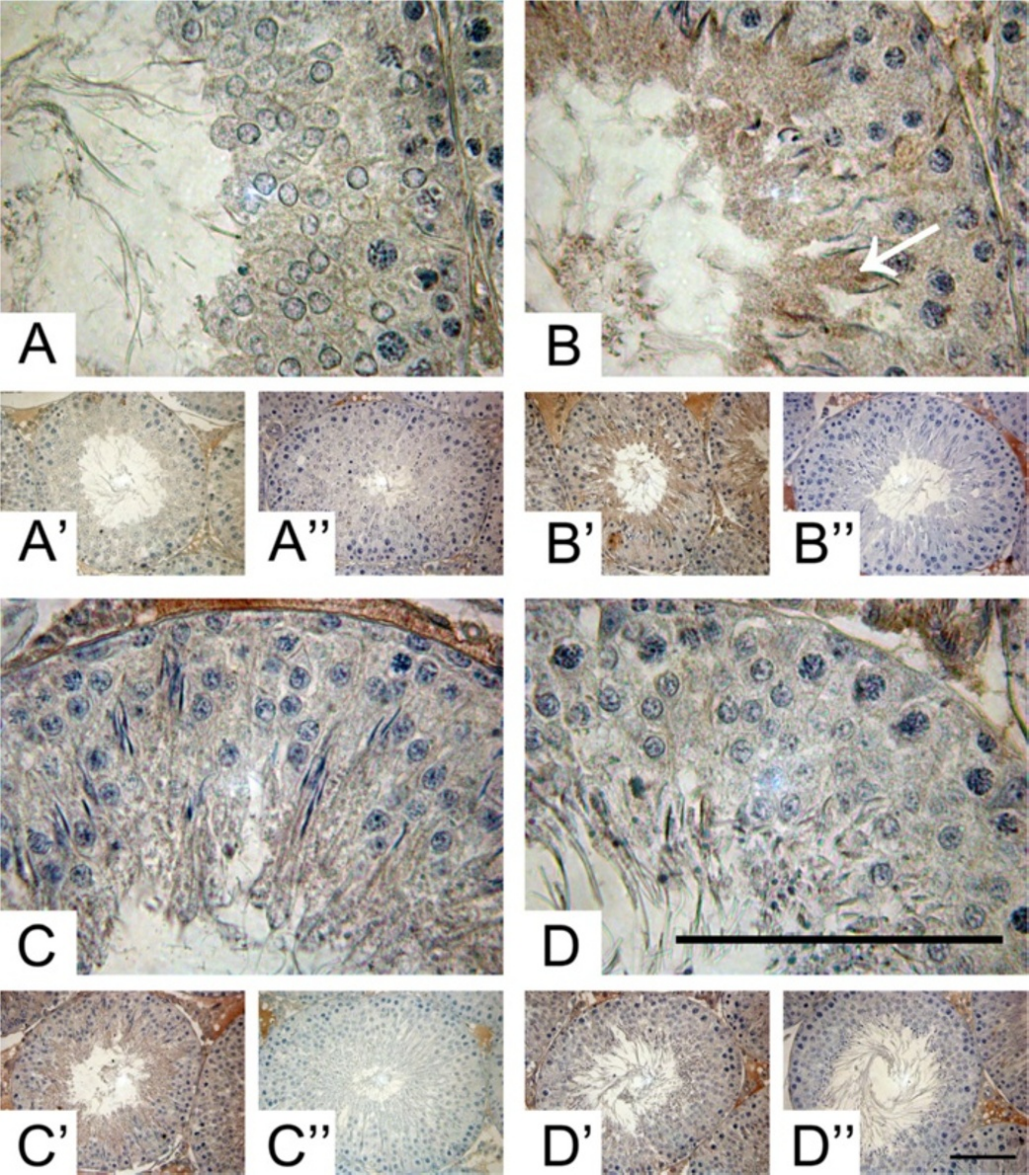
**Stage-specific immunolocalisation of ADAM10 in adult rat testes. (A)** High and low **(A’)** power magnification of Pale (stage IX-XII), Weak (stage XIII-I) **(B, B’)**, Strong (stage II-V) **(C, C’)** and Dark (VI-VIII) **(D, D’)** stages. The label was concentrated in the cytoplasm of elongating spermatids of Weak (stages XIII-I) segments (B, arrow). The negative control, without primary antibody, of the different segments are shown in **A”**, **B”**, **C”** and **D”**. Bars = 50 μm.

**Figure 3:**
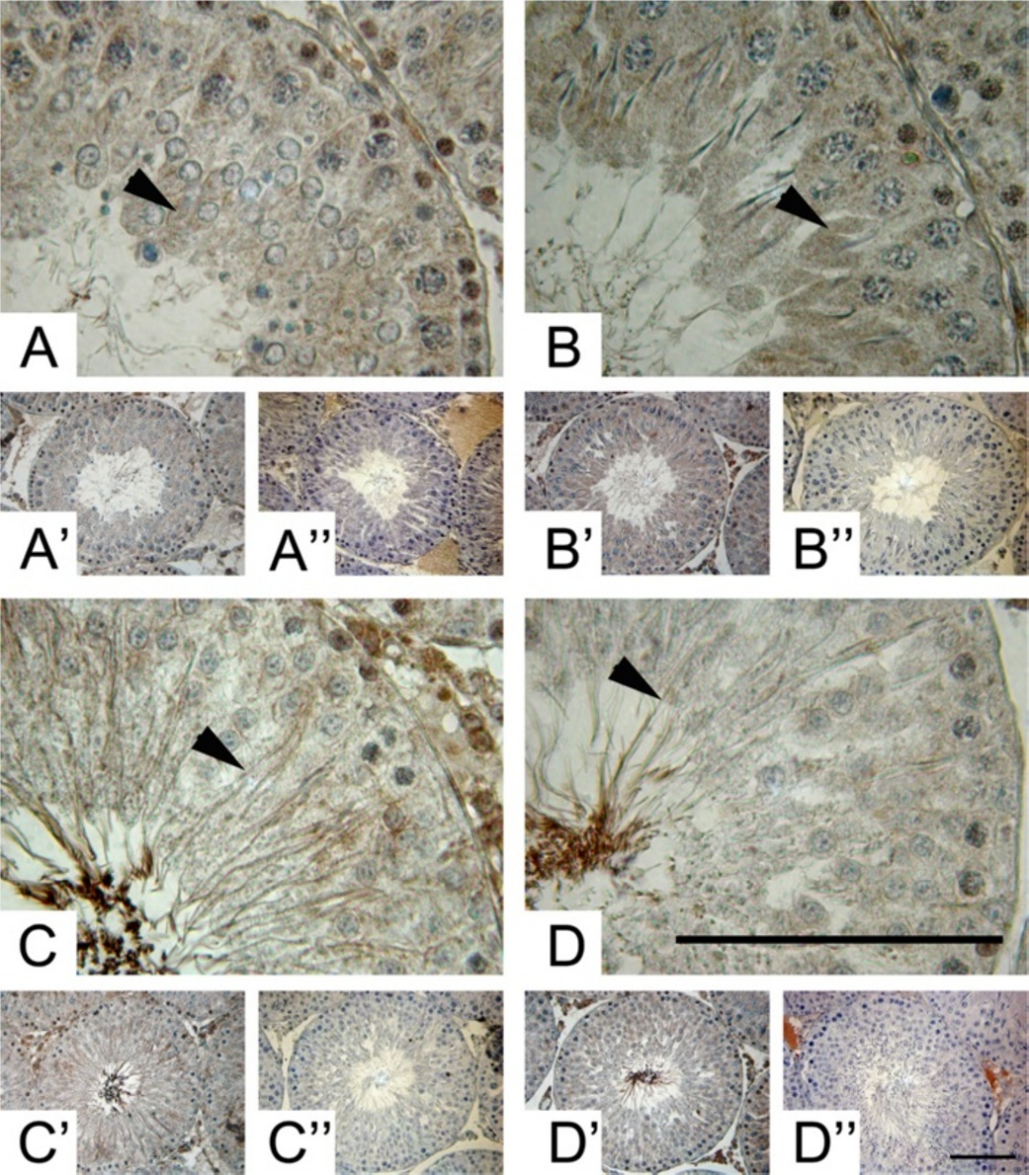
**Stage-specific immunolocalisation of ADAM17 in adult rat testes. (A)** High and low **(A’)** power magnification of Pale (stage IX-XII), Weak (stage XIII-I) **(B, B’)**, Strong (stage II-V) **(C, C’)** and Dark (VI-VIII) **(D, D’)** segments. The residual cytoplasm of elongating spermatids (**A, B,** arrowheads) and the tail of elongated spermatids (**C, D** arrowheads) shows an immunoreaction against the antibody against ADAM17. The negative control, without primary antibody, of the different segments are shown in **A”**, **B”**, **C”** and **D”**. Bars = 50 μm.

**Figure 4:**
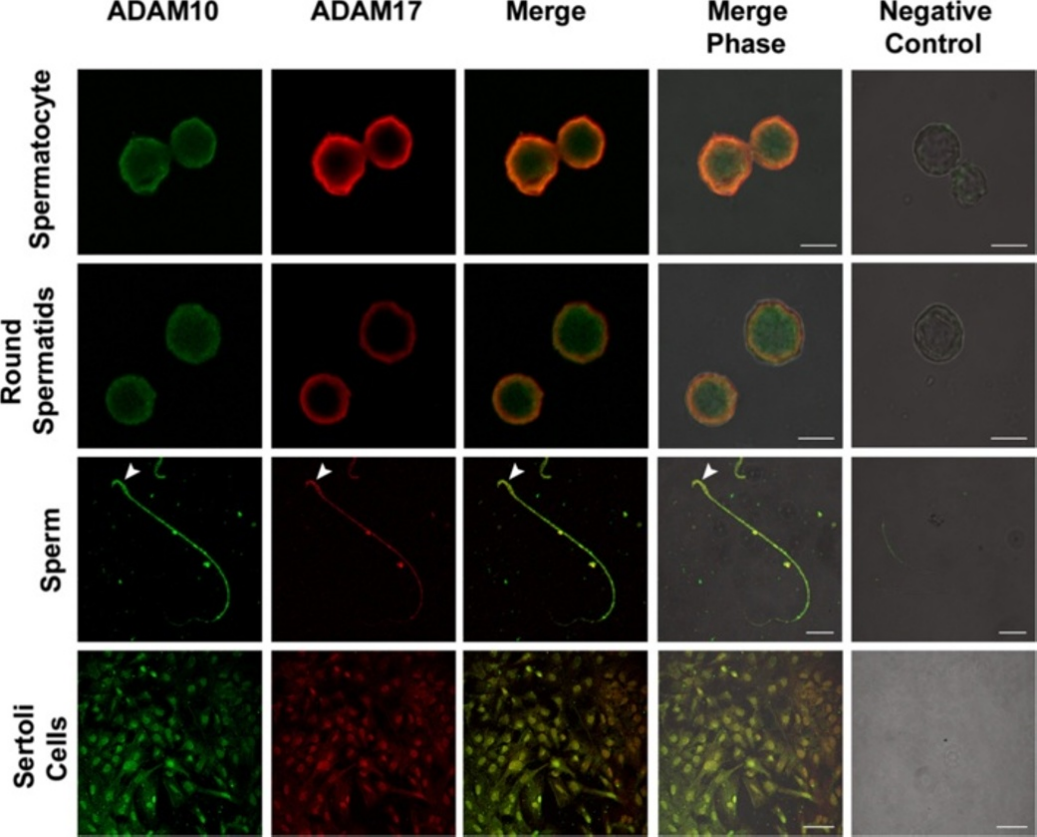
**Colocalization of ADAM10 and ADAM17 in isolated germ and Sertoli cells.** Isolated germ and Sertoli cells were double labelled with anti-ADAM10 (green) and anti-ADAM17 (red). ADAM10 and ADAM17 colocalise in pachytene, round spermatids and Sertoli cells.). Mature caudal epididymis sperm showed immunoreactions against ADAM10 and ADAM17 antibodies in the head (arrowhead) and tail. The last column shows the negative controls (without the primary antibody) merged with the bright field picture. Bars in spermatocyte and round spermatids: 5 *μ*m, sperm: 20 *μ*m, and Sertoli cell: 50 *μ*m.

First we wanted to determine the mRNA and protein levels of ADAM10 and ADAM17 during spermatogenesis. In adult rat testes germ cells are associated in XIV different stages, which can be isolated by using a transillumination-assisted microdissection designed to isolate and characterize specific steps of differentiation [14, 15]. Comparison of the Stages of the Epithelial Cycle Isolated by Transillumination-Assisted Microdissection. This method takes advantage of the differential light absorption of the different stages under the dissecting microscope, and four segments can be isolated: Weak (stage XII–I), the Strong (stage II–V), the Dark (stage VI–VIII) and the Pale (stage IX–XI). This method, in combination with immunohistochemistry is a powerful tool to determine distribution and protein levels of proteins in rat spermatogenesis.Results showed that ADAM10 mRNA levels in Dark segments were significantly higher than Weak segments (Figure 1A,A’) but protein levels were similar in all studied segments (Figure 1C,C’). On the other hand, the mRNA levels of ADAM17 were similar in all segments (stages) (Figure 1B,B’), but its protein levels strongly dropped in Dark as compared with the rest of the segments (Figure 1D,D’). These results suggest that mRNA of ADAM10 and ADAM17 and protein levels of ADAM17 are differentially regulated throughout spermatogenesis.In order to make a better comparison of the biochemical results with those from immunohistochemistry we decide to cluster the different stages of spermatogenesis in the same classification as mentioned above: Pale, Weak, Strong and Dark. Immunolocalization of ADAM10 showed similar immunoreaction intensity in all segments of the seminiferous epithelium (Figure 2), similar to that observed with the protein levels. However, it seems that the label was concentrated in the cytoplasm of elongating spermatids of Weak (stages XIII-I) segments (Figure 2B, arrow). The fact that elongating spermatids are reactive only at stages XIII-I would suggest that steps 13–15 become reactive but not other steps of spermatogenesis. The cytoplasm of Sertoli and germ cells in all the stages of the seminiferous epithelium showed a positive immunoreaction with the antibody against ADAM17 (Figure 3). The immunolabel was weaker in Dark (VI-VIII) than in other segments, which correlated with the protein levels (Figure 1D and Figure 3). Interestingly there was an intense labeling in the nucleus of leptonene (stages IX-XII) and zygotene (Stages XII-XIII) spermatocytes (Figure 3A,B arrows). In addition, a thin label corresponding to the cytoplasm of elongating spermatids was observed in sections of seminiferous tubules corresponding to Strong (stages II-V) segments (Figure 3C, arrow). In addition, the antibody against ADAM10 and ADAM17 gave a detectable signal in isolated germ cells, mature epididymal spermatozoa and Sertoli cells (Figure 4).

**Figure 5:**
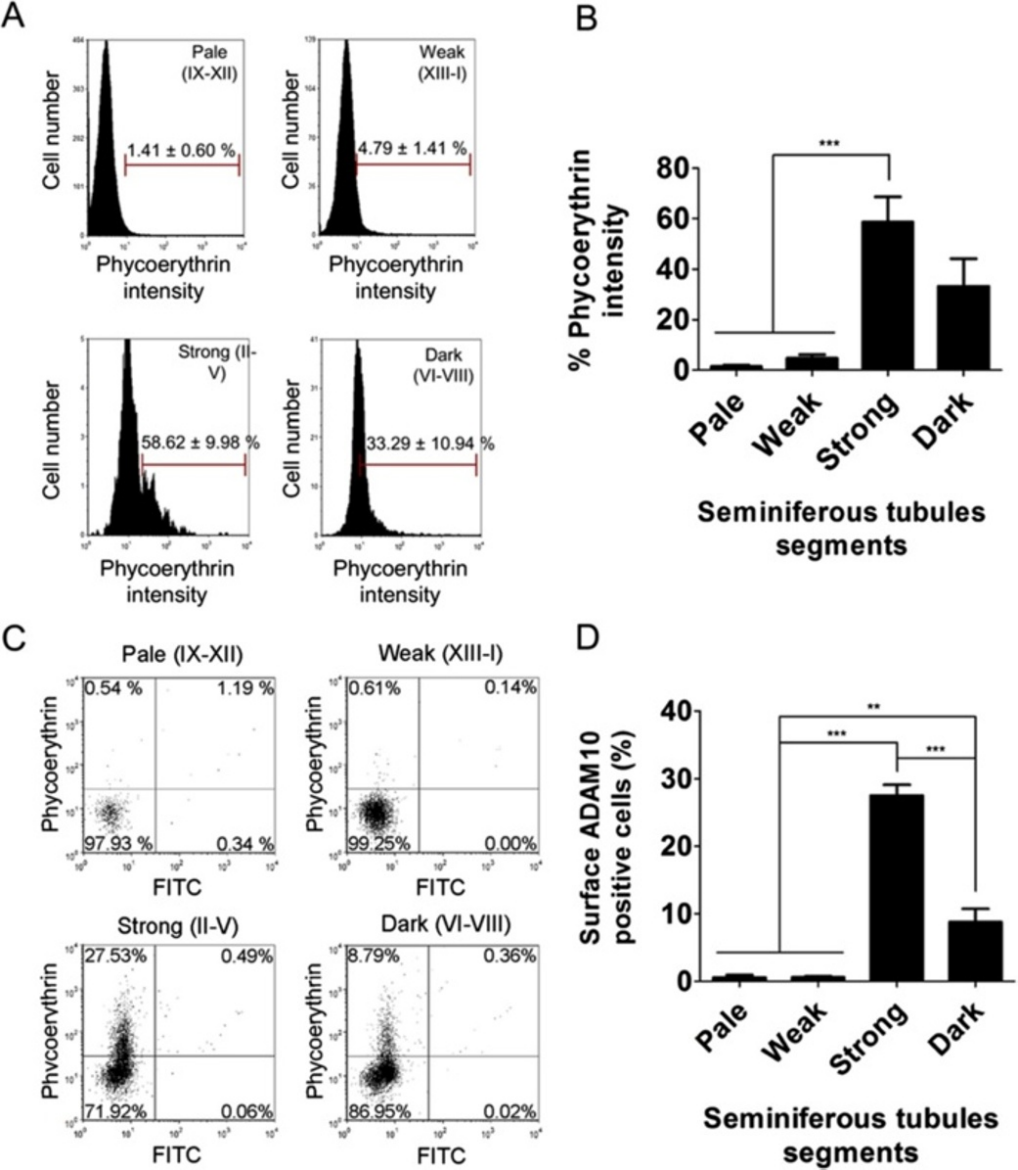
**The amount of ADAM10 at the cell surface and the proportion of cells with ADAM10 at the cell surface are stage-specific.** Seminiferous tubules from adult rat testes were isolated and then dissected according to the different segments (Pale, Weak, Strong and Dark). After disaggregation, the live cells were labelled with the antibodies against ADAM10 coupled to Phycoerythrin. Then, the intensity and the percentage of positive cells were determined by flow cytometry. The figure shows **(A)** the histogram, **(B)** the percentage of fluorescence intensity (amount of ADAM10 at the cells surface), **(C)** the dot plot and **(D)** the percentage of positive cells according to the different segments. The values represent the mean ± S.E.M. of three different experiments (**p < 0.01, ***p < 0.05).

**Figure 6:**
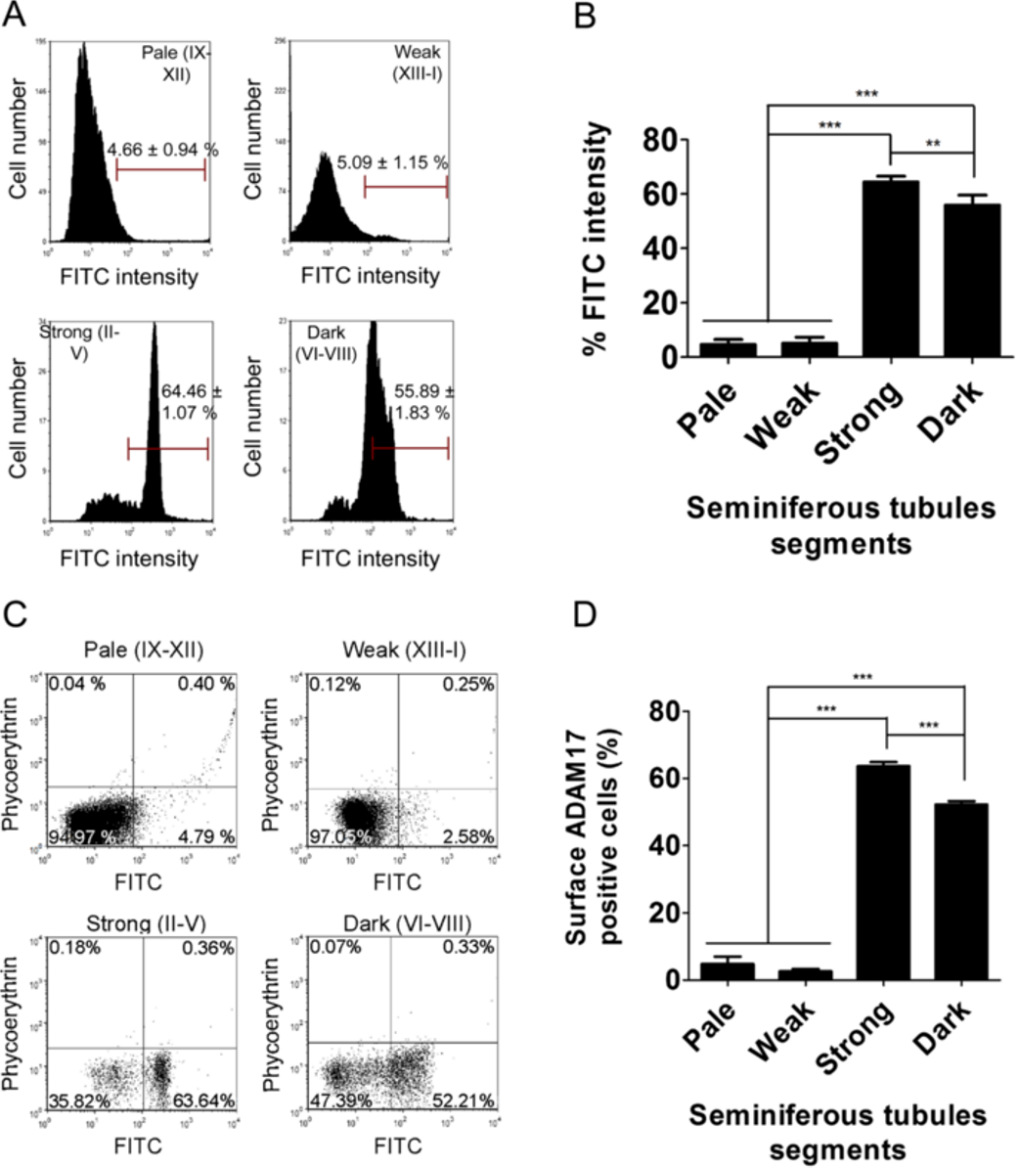
**The amount of ADAM17 at the cell surface and the proportion of cells with ADAM17 at the cell surface are stage-specific.** Seminiferous tubules from adult rat testes were isolated and dissected into the different segments (Pale, Weak, Strong and Dark). After disaggregation, the live cells were labelled with the antibodies against ADAM17 and then with the secondary antibody FITC. The intensity and the percentage of positive cells were determined by flow cytometry. The figure shows **(A)** the histogram, **(B)** the percentage of fluorescence intensity (amount of ADAM17 at the cell surface), **(C)** the dot plot and **(D)** the percentage of positive cells according to the different segments. The values represent the mean ± S.E.M. of three different experiments (**p < 0.01, ***p < 0.05).

ADAM10 and ADAM17 may traffic from intracellular stores to the cell surface under different stimuli, injuries and probably developmental clues. In order to define if their subcellular localization changes during rat spermatogenesis we isolated live cells from the different segments and analyzed the proportion of these proteases at the cell surface by flow cytometry. By analyzing the intensity of the positive cells it was possible to determine if there was any change of total ADAM10 in the different segments. Results showed that the total amount of ADAM10 was similar in Pale and Weak segments, but it robustly increase in Strong and then drop in Dark segments (Figure 5A,B). In addition, the proportion of cells with at the cell surface was similar in cell from Pale and Weak segments, but it robustly increased in strong and then decrease in Dark segments (Figures 5C,D). In the same way, the amount of this protein at the cell surface and the proportion of cells with ADAM17 at the surface was similar in cells from Pale and Weak segments, but it robustly increased in strong and then decrease in Dark segments (Figure 6). Therefore, these results shows that the surface localization of ADAM10 and ADAM17 at the cell surface changes during spermatogenesis.

**Figure 7:**
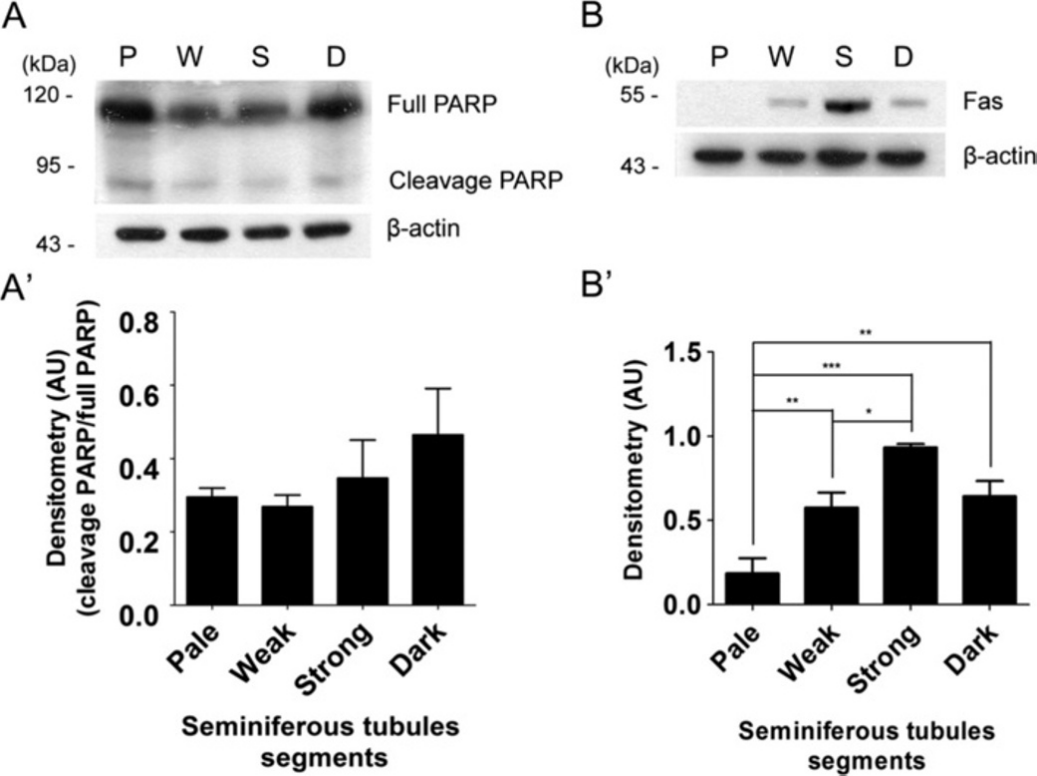
**Stage-specific upregulation of FAS levels. (A, A’)** Poly-(ADP-Ribose) polymerase (PARP) and **(B, B’)** FAS levels in Pale (P, stage IX-XII), Weak (W, stage XIII-I), Strong (S, stage II-V) and Dark (D, VI-VII) segments. There was a tendency of cleaved PARP to increase in Dark segments. FAS levels increased in Weak, Strong and Dark segments as compared with Pale (*, p < 0.0001, **p < 0.01, ***p < 0.05, n = 3). β-actin was used as a loading control.

Finally, we evaluate if some apoptotic markers were coincidently elevated and/or activated with ADAM17 and/or ADAM10. We found that a slights increase of cleavage in the poly-ADP-ribosyl polymerase (PARP), a well know substrate of caspase 3, was observed in Dark and Strong segments (Figure 7A,A’). Fas is a tansmembrane dead receptor that upon binding to its ligans (FasL) or spontaneous trimerization induces apoptosis in many cell types [16–18]. Fas upregulation has been described in germ cell undergoing apoptosis and this leads to activation of the extrinsic pathway of apoptosis [19–22]. Fas was found in Weak and even more abundant in Strong segments, and then decline in Dark segments (Figure 7B,B’). Thus, the upregulation in FAS and cleaved PARP levels in Strong and Dark segments was correlated with cell surface localization of ADAM17 (Figure 7).

## Discussion

Mammalian metalloproteases are involved in cell migration and differentiation in a wide range of cells and tissues. Here we have shown the tissue distribution of ADAM10 and ADAM17 in rat testis in an attempt to find if they are regulated during spermatogenesis.

One intriguing finding was the strong immunolabeling of leptonene (stages IX-XII) and zygotene (Stages XII-XIII) spermatocytes nucleus. Since ADAM17 is a membrane protein that is supposed to be localisated at the Golgi, intracellular vesicles and the cell surface, there is not obvious explanation for this finding. However, it has been found ADAM10 in the nucleus of cancer but not in normal prostate cells, suggesting a role of this particular localization in disease progression [14]. In addition, it has been shown the nuclear localization of several tyrosine kinases receptors and their ligands. In one case, that of ErbB-4, it is processed by a metalloprotease dependent ectodomain cleavage followed by γ-secretase cleavage, within the transmembrane domain and the soluble cytoplasmic fragment it is found in the nucleus [15]. A similar situation could explain the localization of ADAM17 at the nucleus of zygotene spermatocytes.

Since ADAM10 and ADAM17 have not a consensus cleavage sequence the activation of these enzymes has been a matter of controversy. Activation of ADAM17 and ADAM10 are associated with post-traductional modifications of the intracellular domain as well as to the proteolytic processing of the N-terminal by furin at the trans-Golgi network [8, 23–25]. Other experimental evidence have shown that shedding of ADAM17 and ADAM10 substrates occurs at the cells surface suggesting that these enzyme have to traffic from intracellular stores to the cellular membrane in order to meet and shed their substrates [26, 27]. Here we have shown that protein levels of ADAM10 do not change during spermatogenesis, but those of ADAM17 decrease only in stages VI-VIII, suggesting differential regulatory events for these two enzymes during spermatogenesis. This result may suggest that protein levels of this enzyme could be regulated by hormone level, since at this stages the androgen receptor is at the highest levels [28]. Even more interesting, was the finding that the number of cells as well as the amount of ADAM10 and ADAM17 increases at the surface in Strong and Dark segments, which correspond to II-V and VI-VIII, respectively. This result suggests that at these segments these enzymes appear to be activated in order to execute specific tasks during spermatogenesis. In fact, we have previously shown that during the first wave of spermatogenesis, ADAM17 in germ cell undergoing apoptosis and after DNA damage, ADAM17 and ADAM10 are target to the plasma membrane [13]. These results are reinforced by previous reports showing that Phorbol Myristato Acetato (PMA), an inducer of ADAM17 sheddase activity, promotes apoptosis in pubertal and adult testis, suggesting that activation of this enzyme in important for germ cell death [11]. In these cells, the dead receptor Fas is upregulated along with an increased in the cleavage of PARP, a well known caspase-3 substrate. Here we show that in the same segments that ADAM10 and ADAM17 are upregulated at the cell surface, there is an increase in the levels of Fas. This suggests that, as we have shown previously, upregulation of ADAM17 may induce germ cell apoptosis by increasing Fas protein levels at the cell surface [19, 20, 29]. We think that despite that the levels of cleaved PARP do not a stastically significant increase, this value is biologically relevant because it represent the percentage of germ cells is undergoing apoptosis. In addition, Sertoli cells, which do not undergo apoptosis, contribute to mask the extent of germ cell death. Previous reports have shown that physiological apoptosis during adult males is observed in stages XII-XIV and I which correspond to Pale and Weak segments [30, 31]. These data has been collected by detecting *in situ* DNA fragmentation or pycnotic cells, which is a middle-late event in apoptosis. We think that Fas upregulation and ADAM17 cell surface localization are early events in the process of apoptosis induction, which will eventually lead to caspase activation and DNA fragmentation [10]. Thus, this could explain that the stages where dead germ cells are observed are not coincident with those Fas upregulation and ADAM17 cell surface localization. Another possible explanation for these results is that the mechanisms involved during the first wave of apoptosis are different from those during adulthood. In this way, it has been recently shown that apoptotic germ cells induces the release of TNF-α from Sertoli cells, which is a substrate of ADAM17 and ADAM10 [32]. These results reinforce the link between activation of ADAM17 and ADAM10 with germ cell apoptosis in physiological and probably in pathological conditions.

Finally, our histological data show the presence of ADAM10 and ADAM17 in the mature elongating spermatids and spermatozoa. This finding correlates well with previous data showing the inhibition of ADAM17 by a general metalloprotease inhibitor prevents the transactivation of epidermal growth factor receptor (EGFR) during the initiation of acrosome reaction [33]. This results needs to be further confirmed by more biochemical work, but it opens a interesting research avenue.

In summary, here we have shown that the cellular localization of ADAM17 and ADAM10 are developmental regulated during rat spermatogenesis. This is the first report showing a differential cellular localization (intracellular versus surface localization) during differentiation, suggesting that there are specific signal during spermatogenesis that promote localization of ADAM10 or ADAM17 in specific cell compartments.

## Conclusions

The metalloproteases ADAM10 and ADAM17 are expressed in germ and Sertoli cells during rat spermatogenesis. Protein levels of ADAM10 are constant but those of ADAM17 decreased during stages VI-VII. In addition, both enzyme increase their surface localization during specific stages of spermatogenesis. Thus, ADAM10 and ADAM17 are mainly regulated at the post-traductional levels during rat spermatogenesis.

## Methods

### Animals

Male Sprague–Dawley rats were acquired from the Animal Facility of our Faculty. The rats were housed under a 12 L:12D cycle, with water and rat chow being provided *ad libitum*. They were killed by cervical dislocation. Investigations were conducted in accordance with the rules laid down by the Consortium for Developing a Guide for the Care and Use of Agricultural Animals in Agricultural Research and Teaching and by the National Research Council. All animal protocols were endorsed by the Chilean National Fund of Science and Technology (FONDECYT).

### Chemicals and antibodies

Antibodies against ADAM10 (sc- 48400), ADAM17 (sc- 13973), FAS (sc-1023) and PARP (sc-7150) were purchased from Santa Cruz Biotechnology (Santa Cruz, CA). Mouse monoclonal antibody against β-actin (AC-15), rabbit polyclonal antibody against α-tubulin, anti-rabbit IgG-FITC (F0382) and anti-rabbit IgG-TRITC were purchased from Sigma (St Louis, MO). Rat IgG2A anti-ADAM10 conjugated with Phycoerythrin (FAB94GP) was obtained from RD Systems (Minneapolis, MN). Peroxidase anti-mouse IgG, peroxidase anti-rabbit IgG, peroxidase anti-goat IgG and anti-goat IgG-FITC (H + L) antiantibodies were obtained from KPL (Gaithersburg, MD). Anti-mouse and anti-rabbit UltraVision Detection Systems was obtained from Thermo Scientific (Fremont, CA). TRIzol-Reagent and SuperScript II Reverse Transcriptase were obtained from Invitrogen (Carlsbad, CA), *Taq* polymerase Fermentas (Burlington, Canada).

### Isolation and dissection of segments of the seminiferous tubules

Animals were sacrificed by cervical dislocation, and testes were removed, decapsulated and placed in a cooled phosphate buffered saline (PBS) solution [34]. Decapsulated testes were treated with colagenase 0.5 mg/ml in PBS. Isolated tubules seminiferous were washed with cooled PBS and subject to transillumination under the transilluminating dissection microscrope. On basis of their appearance under transillumination the following four principal zones (Pale, Weak, Strong and Dark) were recognized, sectioned and polled for further biochemical analysis [14, 15].

### Isolation of rat pachytene spermatocytes, round and elongated spermatids

Adult rats were sacrificed by cervical dislocation, the testes were removed and the spermatogenic cell population were isolated using velocity sedimentation separation in a 2-4% BSA gradient, as described by Romrell et al. [35]. The pachytene spermatocytes, round spermatids and elongated spermatids were identified by their size as well as by the typical aspect of their nucleous stained with Hoescht_33342_ [36]. From the same rats, cauda sections were separated from the epididymides and placed in 0.3 mL PBS at room temperature under mineral oil. Three incisions were made with fine scissors and the PBS drops containing the tissue were incubated for 30 min to allow the spermatozoa to swim out.

#### Sertoli cells isolation and culture

Sertoli cells were obtained from 17 day old male Sprague–Dawley rats’ testes killed by cervical dislocation. Testes were removed, decapsulated and placed in PBS containing 0.1 mg/ml collagenase (Sigma, St Louis, MO). Then, the tubules were washed three times in PBS. Tubule cells isolation was performed by mechanical disaggregation in the presence of 0.1 mg/ml DNasa (Sigma, St Louis, MO) using a 21G needle from different segments of the seminiferous tubules isolation previously in PBS. Then, the solutions were filtered by through a mesh with a pore of 200 μm and then a pore of 70 μm of diameter. Cells were resuspended in a solution containing PBS and distilled water (1:9) to produce a hypotonic shock, which destroys germ cells but not Sertoli cells. Then, the cells were filtered again, and the filtered solutions were centrifuged for 3 min at 800 × g and resuspended in DMEM-F12 medium without serum and containing only 10% antibiotic and antimycotic (Gibco®, Invitrogen, Carlsbad, CA). 1 × 10^6^ cells were cultured in DMEM-F12 medium supplemented with 10% antibiotic and antimycotic, pH 7.2 at 37°C and 5% CO_2_. After 24 h the cells were washed and cultivated with fresh medium, in the same conditions as above for 4 days, after which, every germ cell is phagocyted by Sertoli cells. The culture obtained has at least 90% purity.

### Immunolocalization of ADAM proteins in tissue sections

ADAM proteins were assayed in paraffin embedded cross-sections of rat testis fixed in Bouin’s solution (Sigma, St Louis, MO, USA). For antigen retrieval slices were treated with sodium citrate 0.01 M, pH 6.0 and heat until boil. The samples were treated with 3% H_2_O_2_ for 10 min, then, to prevent unspecific binding, a standard protein block System (Ultra V block, LabVision, Freemont, VA) was applied for 10 min. Primary antibody against ADAM17 (1 μg/ml) or ADAM10 (1 μg/ml), prepared in 3% BSA in TBS containing 0.1% Tween-20 (TBS-Tween), was applied to the slice and incubated overnight at 4°C in a humidified chamber after being washed three times for 10 min each in TBS-Tween. Biotinylated secondary antibody, streptavidin–biotinylated–peroxidase complex, amplification reagent (biotinyl tyramide) a peroxidase-conjugated streptavidin were applied step by step for 10 min each. Afterwards, slides were washed twice in a buffer for 3 min each. Finally, substrate-chromogen solution consisting of concentrated Tris–HCl and 0.8% H_2_O_2_ (substrate) and 3, 3-diaminobenzidine tetrahydrochloride (DAB) solutions (chromogen) were applied for 5 min and washed in distilled water. Samples were observed under a phase contrast microscope (Zeiss, Germany) and photographed with a digital camera (CoolPix 4500, Nikon, Japan).

### Immunofluorescence of ADAM proteins in isolated germ and Sertoli cells

The localization of ADAM10 and ADAM17 were assayed in isolated germ cells and Sertoli cells fixed in 4% paraformaldehyde in PBS (pH 7.4) 15 min and then permeabilized in cold methanol for 5 min. Then the cells were placed on a slice with 0.1% polylysine. To prevent unspecific binding the samples were treated with 3% BSA in TBS containing 0.1% Tween-20 for 1 h at room temperature. Primary antibodies against ADAM10 and ADAM17 were applied at 1 μg/ml diluted in 3% BSA-TBS containing 0.1% Tween-20 were applied to the slice, which were incubated overnight at 4°C in a humidified chamber, after the slice had been washed three times for 10 min in TBS containing 0.1% Tween-20. Then anti-goat FITC and anti-rabbit TRITC (to ADAM10 and ADAM17, respectively) diluted 1:100 in 3% BSA in TBS containing 0.1% Tween-20 were applied at the slice and they were incubated for 1 h at room temperature, after the slices had been washed three times for 10 min in TBS containing 0.1% Tween-20. Then the slices were mounted with fluoromount (Sigma, St Louis, MO) and were observed under a microscope (Zeiss LSM-510, Germany).

### Cell surface ADAM protein detection

Tubule cells isolation was performed by mechanical disaggregation in the presence of 0.1 mg/ml DNasa (Sigma, St Louis, MO) using a 21G needle from different segments of the seminiferous tubules isolation previously in PBS. Then, the solutions were filtered by through a mesh with a pore of 200 μm and then a pore of 70 μm of diameter. The filtered solutions were centrifuged for 3 min at 800 × g. Then the isolated cells were incubated in DMEM-F12 medium containing 3% BSA, for 30 min at 4°C. Primary antibody against ADAM17, or against ADAM10 coupled to phycoerytrin, was added diluted in blocking solution (1:250) and left to incubate overnight. The next day cells were washed three times with PBS and in the case of those incubated with the antibody against anti-ADAM17 they were dissolved in blocking solution with the corresponding secondary antibody conjugated with FITC (diluted 1:250) and incubated for one hour at 4°C. Then, cells were washed three times with PBS and the final pellet dissolved in PBS. The samples were analyzed by a flow cytometer (FAScanto) and 10,000 gated events were acquired in each sample. As controls, one autofluorescence sample, one sample with only primary antibody and one sample with only secondary antibody were analyzed. All date were analyzed with software FCS express V4.0 (De Novo Software, Los Angeles).

### Protein levels determination

Protein extraction was performed by homogenizing isolated germ cells and Sertoli cells, prepuberal rat testes or seminiferous tubules segments, in buffer A (1% Triton X-100, NaCl 1 M, EDTA 1 mM, PMSF 10 mg/ml, Tris–HCl 20 mM pH 7.0) containing 1 M NaF, 10 mg/ml PMSF, 10 mM orthovanadate, plus a protease inhibitor cocktail (Sigma, St Louis, MO, USA) including 2 mM AEBSF [4-(2-Aminoethyl) benzenesulfonylfluoride hydrochloride], 0.3 μM aprotinin, 130 μM bestatin hydrochloride, 14 μME-64, 1 mM EDTA, 1 μM leupeptin hemisulfatein, and then centrifuging for 10 min at 10,000 × g at 4°C. The supernatant was collected and the samples were run on a 10% polyacrylamide gel (SDS-PAGE) under reducing and denaturing conditions, and then transferred to nitrocellulose at 30 V overnight or 100 V for 1.5 h. Nitrocellulose was blocked with 3% BSA in PBS, pH 7.4, and then incubated overnight at 4°C with anti-ADAM17 (0.2 μg/ml), anti-ADAM10 (0.2 μg/ml), anti-PARP (0.2 μg/ml), anti-Fas (0.2 μg/ml), anti-β-actin (1 μg/ml), or anti-α-tubulin (1 μg/ml) antibodies. After extensive washing with PBS plus 0.1% Tween 20 (PBS-Tween), the membrane was incubated with a secondary antibody conjugated to peroxidase (KPL, Gaithersburg, Maryland) diluted 1:3,000 in PBS-BSA for 1 h at room temperature. Protein bands were revealed using the kit electrochemiluminescence (PerkinElmer Inc, Waltham, USA).

### RNA extraction and RT-PCR

Total RNA of decapsulated testes was isolated using TRIzol-Reagent (Invitrogen, Carlsbad, CA) according to the manufacturer’s recommendations. Total RNA was quantified, and after confirmation of its integrity, cDNA was generated from 1 μg RNA using random primers and SuperScript II Reverse Transcriptase (Invitrogen, Carlsbad). The cDNA obtained was amplified by polymerase chain reaction (PCR) in 30 cycles using Taq polymerase (Fermentas) in 50 μl of the incubation mixture. Several primer sets were used to obtain the PCR products of ADAM10 forward 5′- CCTACGAATGAAGAGGGAC -3′ and reverse 5′-A TCACAGCTTCTCGTGTTCC -3′ ADAM17 forward 5′-GTTGGTGAGCCTGACTCTA-3′ and reverse 5′-CCTCTTGTGGAGACTTGA-3′ GAPDH forward 5′-TCCACCACCCTGTTGCTGTA-3′ and reverse 5′-ACCACAGTCCATGCCATCAC-3′ in the same conditions as previously described. Aliquots of the PCR products were run in a 1% agarose gel and then stained with 0.1 μg/ml ethidium bromide. Bands obtained were analyzed measuring the pixels with Adobe® Photoshop 7.0 (Adobe System Incorporated, USA), and normalized to GAPDH mRNA levels.

### Statistical analysis

For mean comparisons, we used analysis of variance (ANOVA). When the ANOVA test showed statistical differences, the Tukey post test was used to discriminate between groups. Statistical significance was defined as p < 0.05 (Sokal, 1995). Statistical analyses were performed using GraphPad Prism version 5.0 for Windows (GraphPad Software, San Diego California USA, http://www.graphpad.com).
